# Subjectivity associated to the use of rock mass classification in stability analysis of caverns

**DOI:** 10.1038/s41598-025-05055-4

**Published:** 2025-07-19

**Authors:** Sailesh Adhikari, Krishna Kanta Panthi, Chhatra Bahadur Basnet

**Affiliations:** 1 Norwegian University of Science and Technology, Trondheim, Norway; 2 Tribhuvan University, IOE, Pashchimanchal Campus, Pokhara, Nepal

**Keywords:** Geological Strength Index (GSI), Rock mass classifications, Deformation in the cavern, Stability analysis, Numerical modelling, Civil engineering, Power stations

## Abstract

Q and RMR systems of rock mass classifications are widely used around the world to characterize rock mass quality and to estimate preliminary rock support for underground structures like tunnels and caverns. Despite being widely used, the ratings given in these classification systems are highly subjective and are based on the judgment of the site engineers and engineering geologists. While carrying out such characterization, parameters associated with each classification system are reported in a range of values instead of a single value. On the other hand, in recent times, Geological Strength Index (GSI) has been used extensively worldwide while carrying out stability assessments of tunnels and caverns. Through this process, the GSI value is linked to different relationships proposed by different scholars. This manuscript aims to demonstrate the sensitivity of the variation of the rock mass quality ratings and their impact on the assessment of the overall stability condition of underground caverns. The in-depth assessment of the deformation condition is analyzed using numerical modelling for different GSI values from the same rock mass class classified by Q-system. For this purpose, an underground powerhouse cavern located in the higher Himalayan region is considered a case project. The powerhouse cavern is characterized by Q-system values ranging from 3 to 40 and GSI values from 44 to 73. Stability assessments are carried out using combinations of numerical, empirical, analytical, and semi-analytical approaches. The analysis indicated that the cavern remains stable, but the results exhibited notable variation due to the sensitivity of GSI as an input to the analyses. Finally, the limitations of the use of GSI in numerical modelling are comprehensively discussed.

## Introduction

Rock mass classification is widely used in the planning, design, and construction of underground structures like tunnels and caverns. Rock mass classifications quantify the quality of the rock mass and are useful tools for engineers for the development of underground space. The surface mapping, sub-surface investigations, and laboratory tests of the rock samples are the important features that describe the rock and rock mass properties^1^. However, the established parameters are subjective and increase uncertainties in almost all types of rock mass classification systems^2^. The widely used rock mass classifications are the Q-system^3^Rock mass rating (RMR)^4^and Geological Strength Index (GSI)^5^. According to Hoek and Brown (1997)^6^GSI provides a numerical value for estimating the rock mass characteristics based on the surface quality and interlocking of rock pieces in the rock mass. GSI assessment is subject to visual inspection by the site engineer or geologist, which may end with different results for the same rock mass. To overcome this limitation, giving a range of values rather than a single value of GSI is suggested. Thus, the reliability of the GSI estimate depends upon the experience gained by the site personnel. Q system expresses the rock mass quality based on the six parameters. These parameters are related to the size of intact rock blocks in the rock mass, shear strength along the discontinuity planes, and the stress environment of the rock blocks. Theoretically, Q values lie between 0.001 for exceptionally poor rock mass to 1000 for exceptionally good rock mass^3^. RMR system provides the rating for six different parameters based on the UCS, RQD, joint spacings, discontinuity conditions, groundwater inflow, and orientation (alignment) conditions. The quality condition of the rock mass is defined from very good quality (RMR rating 100 − 80) to very poor quality (RMR rating less than 20). Due to the range of quantitative values of different parameters of rock mass classification, the result obtained may vary considerably.

It is of utmost importance to apply the rock mass classification with high caution for the design and construction of tunnels and caverns. An additional input from engineering observation and judgment of real ground conditions is necessary for the rock support design. The correlation of the RMR and Q system in the Nepal Himalayas was studied by Chaulagai and Dahal (2023)^7^ and it was found that the correlation trend follows the ranges of the existing relationship between Q and RMR proposed by various researchers. Pozo (2022)^8^did a qualitative and quantitative analysis of GSI and RMR in various rock mass qualities and suggested that care must be given while applying GSI to the poor and very poor-quality rock mass. For the tectonically disturbed rock mass with destroyed structural fabric, the quantitative GSI is not effective, and a qualitative GSI based on visual observations must be used^9^. RMR is dependent upon the RQD, which may be zero for the weak rock mass, hence, the applicability of RMR is suitable for reasonable quality rock mass (30 < RMR < 70)^10^. Palmstrom and Broch (2006)^11^ have suggested that the Q system only works best for the Q values in the range of 0.1 to 40, span/ESR ratio varying from 2.5 to 30, and the underground structures in hard and jointed rock mass having no overstressing. GSI applies only to weak and homogeneous rock mass with either no discontinuity or heavily jointed rock mass^12^. As all rock mass classification systems have their limitations, rock engineers and engineering geologists must not rely on a single classification system but use more than one and use the ranges of values highlighting typical mean, maximum, and minimum values.

The Hoek-Brown (HB) failure criterion was initially developed by Hoek (1965)^13^and it was further developed for the jointed rock mass by Brown (1970)^14^(Hoek and Brown, 2018)^15^. GSI introduced by Hoek (1994)^5^ was linked to the HB failure criterion. Since then, it has been widely used to estimate the strength and deformation properties of heavily jointed and weak rock mass^15^. The generalized HB failure criteria are expressed by Eq. 1. The rock mass parameters associated to the failure criteria are being estimated using relationships given by Eqs. 2–4.

1 $$\:{\sigma\:}_{1}=\:{\sigma\:}_{3}+{\sigma\:}_{ci}{\left({m}_{b}\frac{{\sigma\:}_{3}}{{\sigma\:}_{ci}}+s\right)}^{a}$$

2 $$\:{m}_{b}=\:{m}_{i}{e}^{\left(\frac{GSI-100}{28-14D}\right)}$$

3 $$\:s={e}^{\left(\frac{GSI-100}{9-3D}\right)}$$

4 $$\:a=\:\frac{1}{2}+\frac{1}{6}\left({e}^{-GSI/15}-{e}^{-20/3}\right).$$

Where, *σ*_*1*_ and *σ*_*3*_ are the major and minor principal stresses, respectively; *σ*_*ci*_ is the unconfined compressive strength of intact rock; *D* is the disturbance factor subjected to blasting damage and is dependent on the quality of excavation; *m*_*b*_, *s*, and *a* are the rock mass material constants,

The material constant parameters *m*_*b*_, *s*, and *a* for the rock mass depends on the values of *GSI* and *D*, which are highly subjective. The value of *D* ranges from 0 to 1, where 0 indicates no disturbance in the tunnel wall due to blasting and 1 indicates excessive damage to the tunnel wall due to poor blasting conditions. Tunnels excavated using TBM give zero value of *D*, on the other hand, the precise values of D for the tunnels and caverns excavated using drill and blast are challenging to estimate. Similarly, the estimation of *GSI* value is also very subjective, hence, the estimation of the rock mass material constants given in the above equations has uncertainties. The material constant of intact rock *m*_*i*_ is dependent upon the rock type and texture of the rock. Hoek (2007)^16^ has tabulated the values of *m*_*i*_ for different rocks; the use of maximum and minimum values in ranges of the suggested values alters the rock mass parameters, indicating sensitivity in the assessment. Apart from the guideline provided in tabular form, *m*_*i*_ can also be estimated from Hoek (2007)^16^ equations and rigidity approach, which are discussed later in this manuscript.

The value of GSI plays a crucial role in estimating the mechanical behaviour of rock mass, especially when the intact rock properties alone are insufficient for the design of underground structures. Its primary mechanical significance lies in estimating the rock mass strength and computing the deformation modulus. The major drawback of GSI is that it assumes homogeneity and treats the rock mass as uniformly fractured. In reality, rock mass often consists of joints, foliation planes influenced by shearing and weathering, which significantly change the structure and material properties of rock mass over short distances. Additionally, GSI doesn’t provide direct correlations with the support systems, and also doesn’t explicitly account for the influence of groundwater conditions, even though these can significantly affect rock mass behaviour.

Considering this fact, the manuscript evaluates and discusses the sensitivity in the use of GSI as a tool to characterize rock mass quality and deformation extent for the powerhouse cavern of the Super Dordi Hydropower Project-Kha (SDHP), Lamjung, Nepal. The analysis utilizes the Q-system of rock mass classification used at the site to estimate GSI values empirically. Further, the GSI values are used in numerical simulations to assess the deformation in the periphery of the powerhouse cavern. The laboratory test results are used as input parameters for numerical modelling. The triaxial test results are used to validate the empirical relationship for determining Mohr-Coulomb (MC) parameters based on GSI. The advantages and limitations of employing GSI in numerical modelling assessment are discussed.

## Methodological frameworks, case description, and GSI estimation

### Methodology

**Fig. 1:**
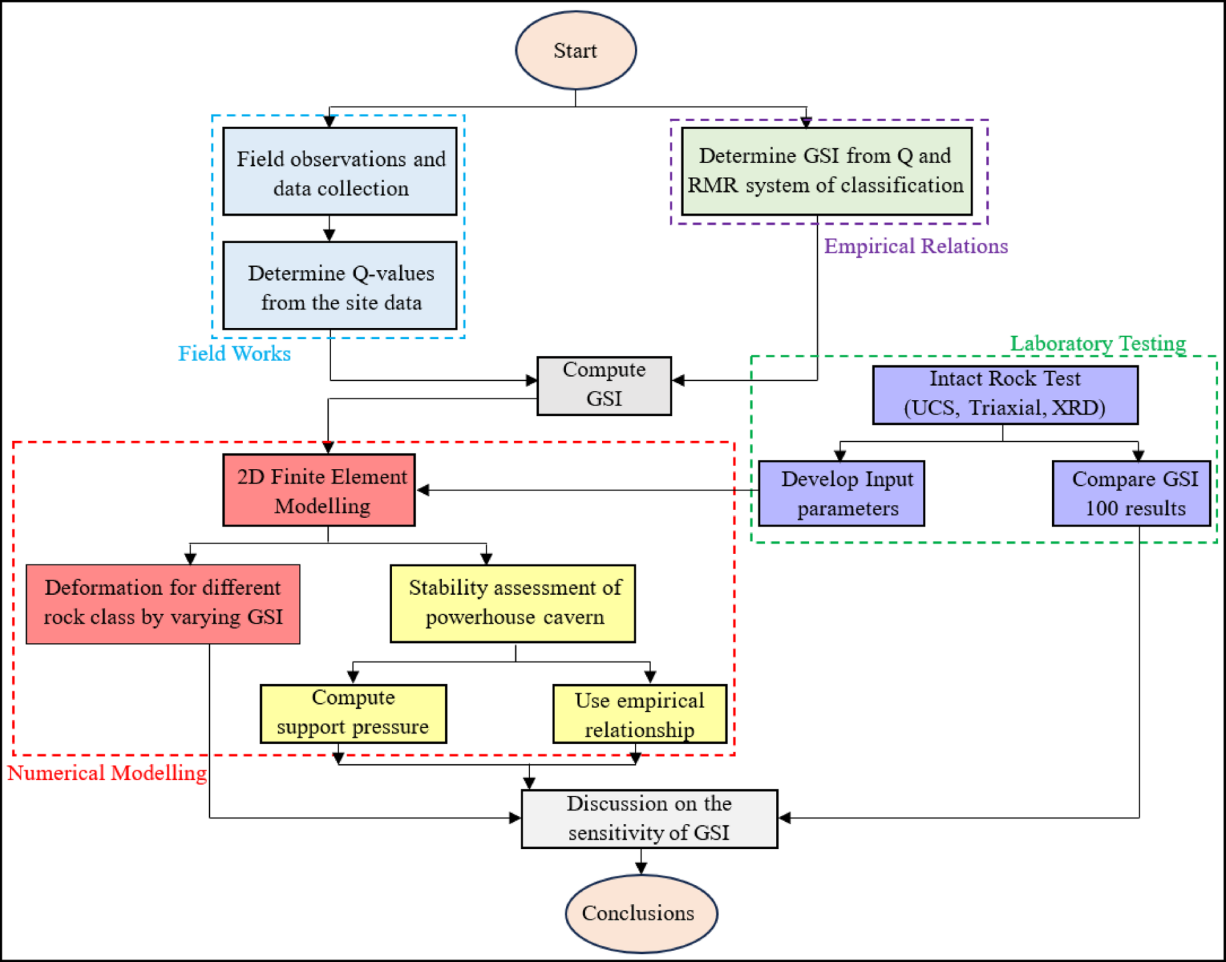
Illustration of the research methodology for the sensitivity analysis of GSI.

The approach to sensitivity assessment of GSI is illustrated in Fig. 1. It includes a combination of fieldwork, laboratory testing, numerical modelling, and use of empirical relationships. Initially, the field data are collected to establish the ranges and typical mean for all six parameters of Q-value. Then the minimum and maximum Q-values are determined. The ranges of Q-values characterize the rock mass into different quality classes. Using the empirical relationship, the GSI is obtained in a range from the Q-values, which is further used in the sensitivity analysis. The laboratory test of the intact rock is done to establish mechanical properties, which are used as input parameters for numerical modelling. Subsequently, triaxial test results are utilized to compare with the empirical relations obtained for a GSI of 100. 2D finite element models are prepared using RS^2^developed by Rocscience (2022)^17^to compute the deformation for various GSI values. Finally, the results are discussed in terms of GSI sensitivity, and conclusions are drawn.

### Brief description of case project

**Fig. 2:**
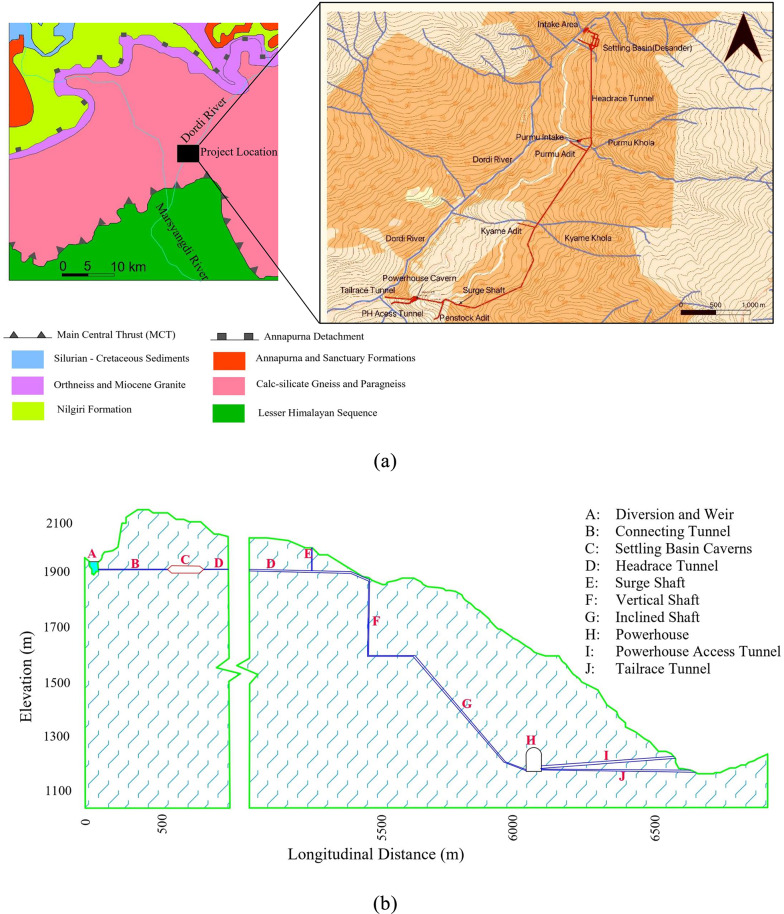
Geological details and components of SDHP; (**a**) regional geological and topographic maps (**b**) longitudinal section with major structural components.

The case project for this manuscript is the Super Dordi Hydropower Project-Kha (SDHP), which is a run-of-river type hydropower project with an installed capacity of 54 MW. The project has a gross head of 638 m. As seen in Fig. 2a, the project lies in the lower boundary of the higher Himalayan rock formation and is about 10 km north of the Main Central Thrust (MCT). Figure 2b shows the major structural components of SDHP. The project site is characterized by medium to high-grade metamorphic rocks consisting of schist and gneiss. The underground powerhouse cavern is situated in gneiss rock, which is schistose and consists of mica minerals along the schistosity planes. The rock mass is of moderate strength and displays moderately brittle behavior. The rock mass is influenced by tectonic shearing since the cavern is located at a relative proximity to the Main Central Thrust (MCT). The feasibility study indicated that the project has no significant risk associated with geological conditions^18^. Initially, the powerhouse was planned at the surface instead of underground. However, during the Gorkha earthquake in April 2015, there was a minor landslide near the selected powerhouse site’s uphill area, making it risky to build the powerhouse on the surface. Eventually, it was decided to build an underground powerhouse instead of the surface powerhouse^19^. During construction, a shear band approximately 10 to 20 cm thick was encountered near the initially proposed location of the underground powerhouse cavern. The shear band, likely associated with localized tectonic deformation and weakness zones within the rock mass, posed a potential risk to cavern stability. To mitigate this, the cavern location was shifted approximately 40 m further into the mountain to reduce the influence of this shear zone to the in-situ stress condition. As expected, the rock mass encountered in the powerhouse cavern area exhibited good quality. Also, the powerhouse cavern is aligning favorably regarding major joint sets^20^ and is designed to avoid major fault zones. The rock mass was found to be relatively dry in the powerhouse area, with exceptions in the locations where minor dripping of water was seen, resulting in localized dampness in a few locations of the cavern. Thus, the site location and geological conditions have been good regarding the stability of the powerhouse cavern.

**Fig. 3:**
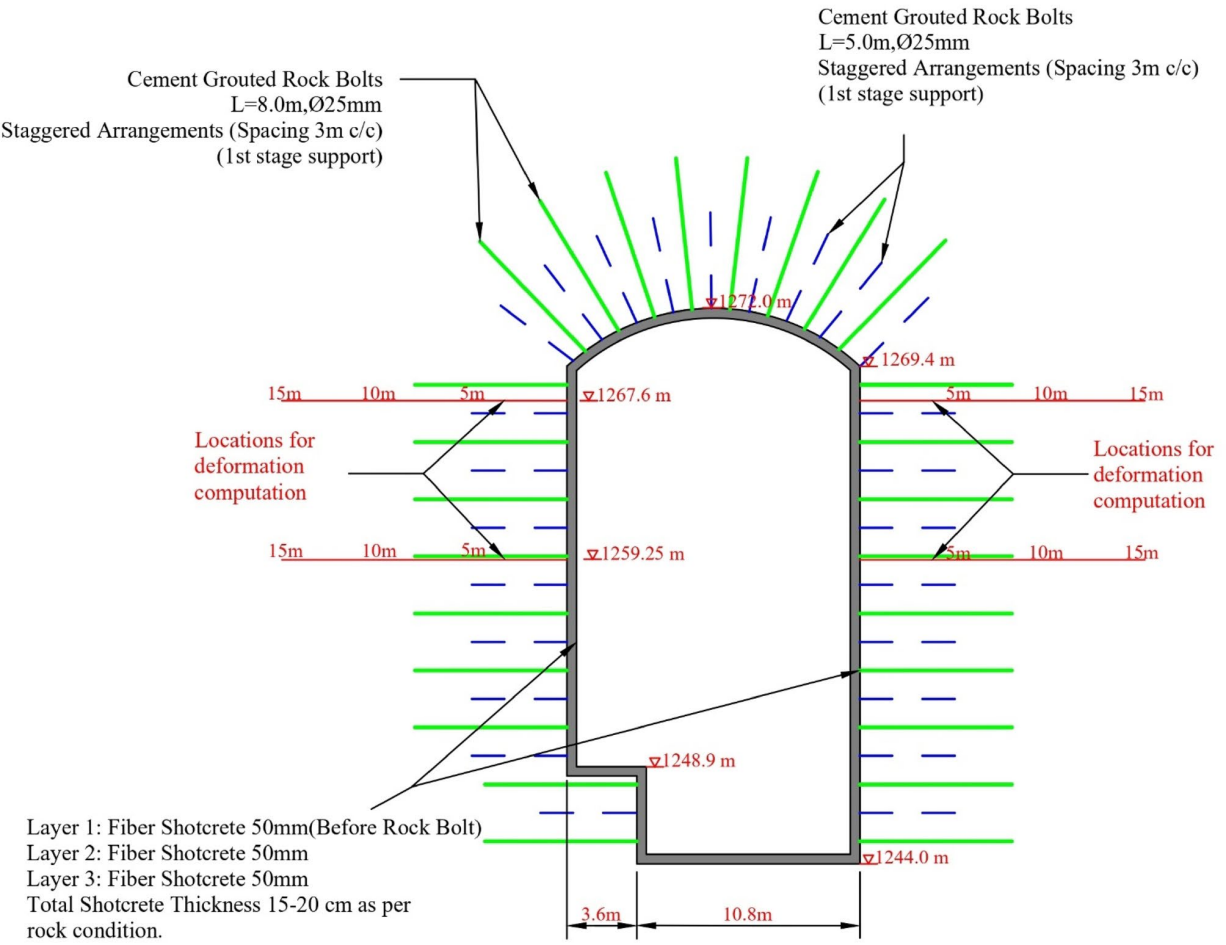
Cross section of the powerhouse cavern with support details and locations of deformation computation.

The powerhouse cavern has an inverted D-shape and has dimensions: 39 m long, 14 m wide, and 28 m high. Long-term creep deformation in the powerhouse cavern is monitored and recorded using multi-point borehole extensometers installed at four different locations of the cavern. Figure 3 shows the cross-section of the powerhouse cavern with the applied support and location of deformation measurement. As initial support, 50 mm fiber-reinforced shotcrete (layer 1) was provided, followed by the installation of 5 m long rock bolts of 25 mm diameter at a spacing of 3 m in a staggered arrangement. Another layer of 50 mm fiber-reinforced shotcrete (layer 2) was provided after the installation of rock bolts. Cement-grouted rock bolts of 8 m length and 25 mm diameter at a staggered arrangement with a spacing of 3 m c/c were provided just before layer 3 of fiber-reinforced shotcrete of 50 mm thickness (Fig. 3). Hence, the final shotcrete thickness in the cavern varies between 15 and 20 cm depending upon the rock mass quality condition. The applied support serves as the final support.

### GSI estimations

The rock mass quality assessment was made using Q system of rock mass classification at the powerhouse cavern. From the field mapping records, the typical mean for the six Q parameters and their ranges for the powerhouse cavern are summarized in Table 1. In this article, GSI is determined using both empirical relationship (Eq. 5) proposed by Hoek et al. (2013)^21^ and direct rock mass quality observation in the caverns.

**Table 1:** Rock mass quality of powerhouse cavern location.

Parameters	Symbol	Value	
		Range	Typical mean
Rock quality designation	*RQD*	60–80	70
Joint set number	*J*_*n*_	3–6	4
Joint roughness	*J*_*r*_	1.5–3	2
Joint alteration	*J*_*a*_	2–4	3
Joint water reduction	*J*_*w*_	0.8–1	0.9
Stress reduction factor	*SRF*	1	1
$\:Q=\frac{RQD}{{J}_{n}}\times\:\frac{{J}_{r}}{{J}_{a}}\times\:\frac{{J}_{w}}{SRF}$		40 − 3	10.5

The parameters presented in the table give a minimum Q-value of 3, and a maximum Q-value of 40, which indicates that the powerhouse cavern has a dominant rock mass class consisting of fair to good quality and has a typical mean Q-value of 10.5.

5 $$\:GSI=\:\left(\frac{52\left({J}_{r}/{J}_{a}\right)}{1+{J}_{r}/{J}_{a}}\right)+\frac{RQD}{2}.$$

Equation 5 gives GSI values ranging from 44 to 72, which resemble fairly with the quality conditions seen in the cavern. These ranges of GSI values are considered to compute the deformation in the cavern.

## Assessment of input variables

### Intact rock properties

**Fig. 4:**
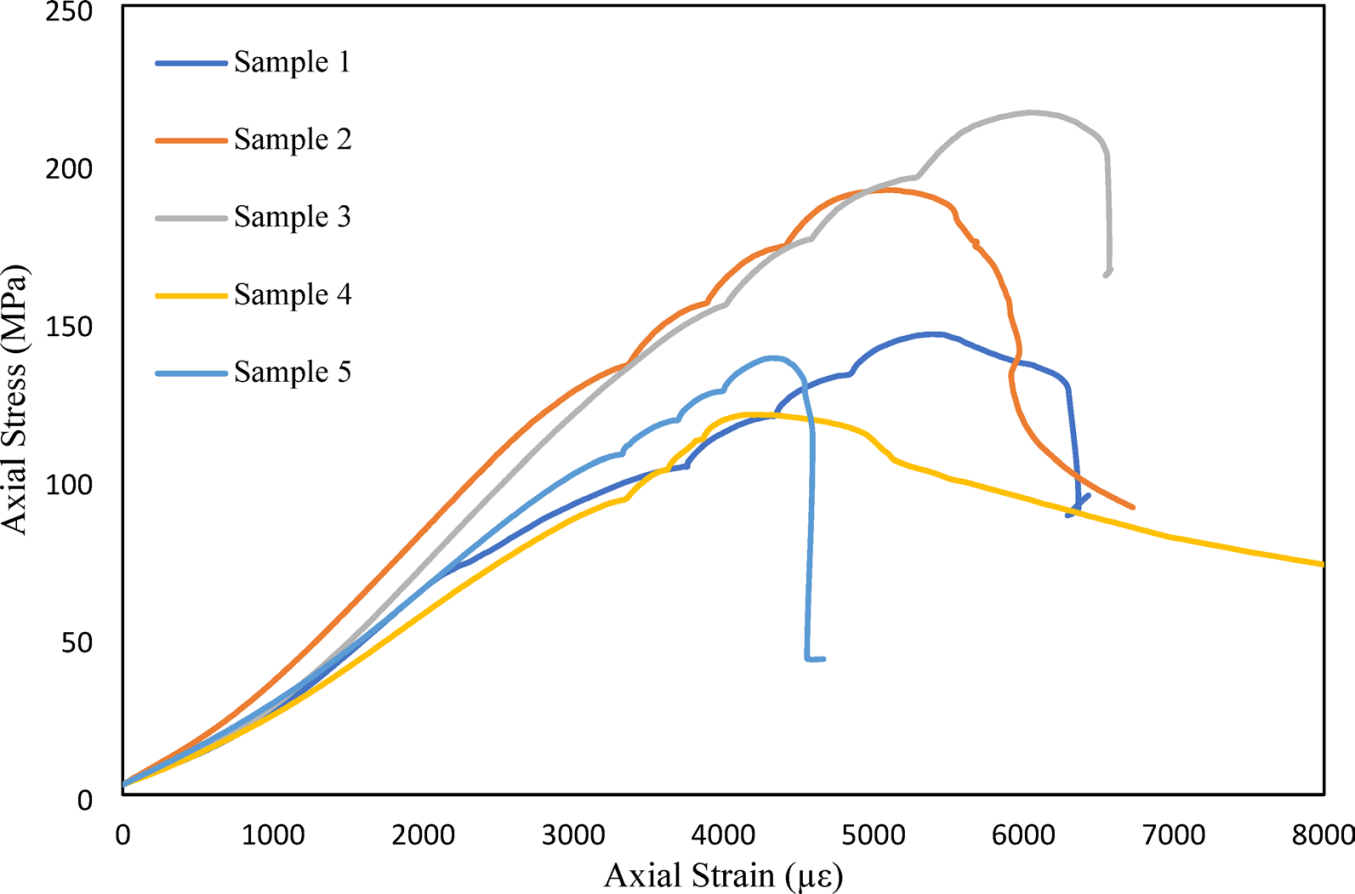
Triaxial Testing of core samples at different confining pressures.

The laboratory testing of the rock samples from the SDHP powerhouse cavern was conducted at NTNU rock engineering laboratory. The core samples were collected from multiple locations and varying depths of excavation in the cavern to represent the overall condition of the cavern. The laboratory testing of rock samples gave an average intact rock strength (UCS) of 112.7 MPa with a standard deviation of 10.4 MPa. Young’s modulus and Poisson’s Ratio of the intact rock were found to be 48.53 GPa and 0.33, respectively. XRD test revealed the major mineral composition as quartz (≈ 58%), feldspar (≈ 24%), and mica (≈ 16%), respectively. The multi-stage triaxial test was also performed with the confining pressure variations of 2 MPa, 4 MPa, 6 MPa, and 8 MPa on the five intact rock samples, respectively. The confining pressure range has been selected to represent the combined effects of tectonic and gravitational stresses acting on the cavern. This range is also used to cross-check the approximate intact rock strength at the powerhouse location. Figure 4 illustrates the triaxial test envelopes obtained from five different rock core samples.

The triaxial test results and Eq. 6 proposed by Hoek (2007)^16^ were used to calculate the intact rock strength of 107.25 MPa.

6 $$\:{\sigma\:}_{ci}^{2}=\frac{{\sum\:}_{i=0}^{n}y}{n}-\left[\frac{{\sum\:}_{i=0}^{n}xy-\left({\sum\:}_{i=0}^{n}x{\sum\:}_{i=0}^{n}y/n\right)}{{\sum\:}_{i=0}^{n}{x}^{2}-\left({\left({\sum\:}_{i=0}^{n}x\right)}^{2}/n\right)}\right]\frac{{\sum\:}_{i=0}^{n}x}{n}.$$

Where, *x* is the value of confining stress (*σ*_*3*_), and *y* is the square of the difference between the principal stresses, i.e. *y=* (*σ*_*1*_ *- σ*_*3*_)^2^*n* is the number of test results. The intact rock strength was also computed from the MC envelope, which indicated a value of 110.45 MPa. Since the intact rock strength from the three approaches is close to each other, an average value of 112 MPa, obtained from the UCS test, is used for the analysis.

The term *m*_*i*_ is the indicator of the brittleness of the intact rock and is constant. The value of *m*_*i*_ is influenced by mineral composition, foliation, grain size (texture), cementation, etc^22^. Hoek (2007)^16^ recommends a table to roughly estimate *m*_*i*_ values for the different types of rocks, which is often used when the test data are unavailable. In addition, other approaches exist to estimate the value of *m*_*i*_, such as the Equation proposed by Hoek (2007)^16^, the rigidity index approach^23^, etc.

From the Hoek (2007)^16^ table, the *m*_*i*_ value for the Gneiss is 28 ± 5. Similarly, Eq. 7 proposed by Hoek (2007)^16^ uses triaxial test results to calculate the value of *m*_*i*_. In equation *x*, *y*, and *n* are the same as explained above. Equation 7 gave the value of *m*_*i*_ as 19.32.

7 $$\:{m}_{i}=\frac{1}{{\sigma\:}_{ci}}\left[\frac{{\sum\:}_{i=0}^{n}xy-\left({\sum\:}_{i=0}^{n}x{\sum\:}_{i=0}^{n}y/n\right)}{{\sum\:}_{i=0}^{n}{x}^{2}-\left({\left({\sum\:}_{i=0}^{n}x\right)}^{2}/n\right)}\right].$$

The rigidity approach proposed by Cai (2010)^23^ to compute *m*_*i*_ is given in Eq. 8. It considers the laboratory test results of the tensile strength and compressive strength of the intact rock. The tensile strength test carried out at the lab following ISRM (1978)^24^ suggested method gave tensile strength of 5.83 MPa with a standard deviation of 1.35 MPa. The calculation made using Eq. 8 gave *m*_*i*_ values ranging from 15 ± 7.

8 $$\:\frac{{\sigma\:}_{c}}{\left|{\sigma\:}_{t}\right|}=0.81\:{m}_{i}+7.$$

Since the *m*_*i*_ value calculated using Eq. 7 falls between the lower bound of values suggested in the table and the upper bound of Eq. 8, the value of 22 is adopted in this research.

### Rock mass compressive strength and deformation

The compressive strength of rock mass and deformation modulus are influenced by various factors like in-situ stress, strength anisotropy, discontinuity, groundwater conditions, weathering, etc^2^. There are various relationships to estimate the deformation modulus and compressive strength of rock mass. The compressive strength of the rock mass and deformation modulus for the powerhouse cavern are estimated using Eqs. 9^25^ and 10^26^, respectively.

9 $$\sigma _{{cm}} = \sigma _{{ci}} \cdot \user2{s}^{a}$$

10 $$\:{E}_{rm}={E}_{i}\left(0.02+\frac{1-D/2}{1+{e}^{\left(\left(60+15D-GSI\right)/11\right)}}\right).$$

**Fig. 5:**
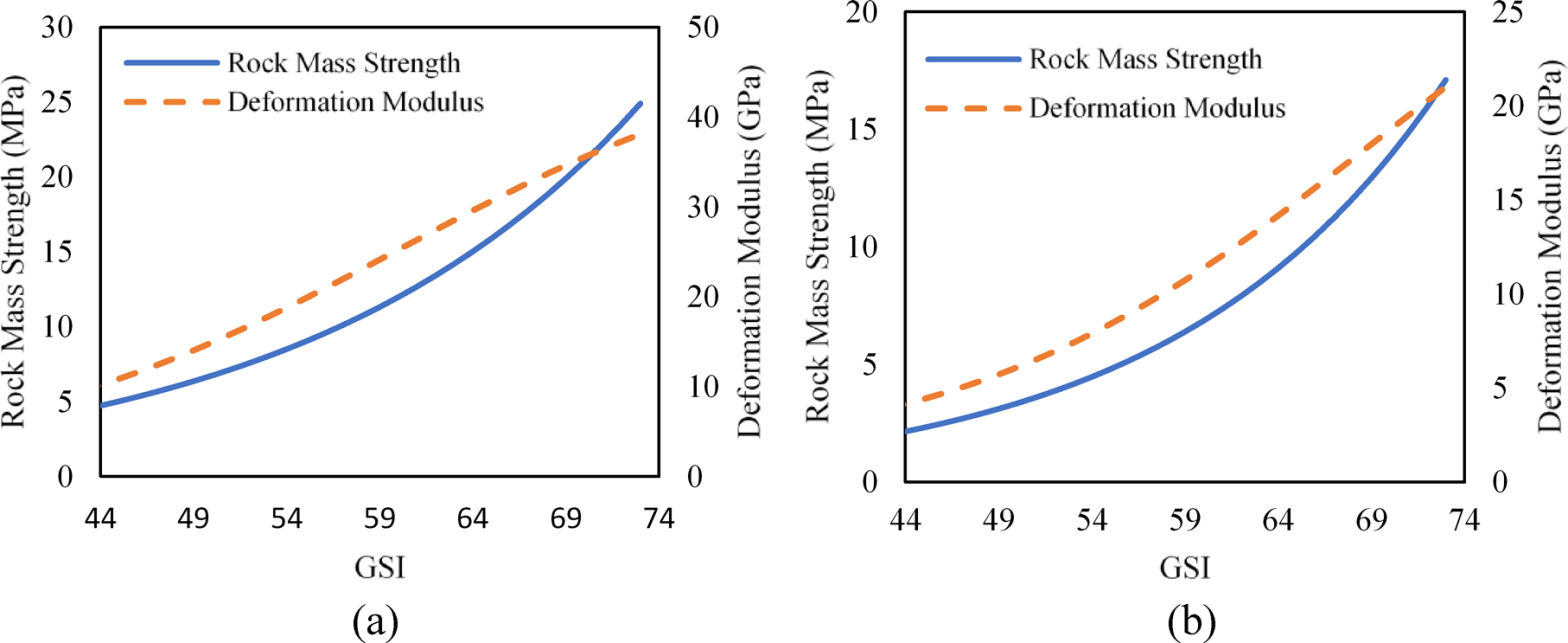
Compressive strength of rock mass and deformation modulus for the (**a**) undisturbed zone (**b**) disturbed zone.

Where, *σ*_*cm*_ is the compressive strength of rock mass, *σ*_*ci*_ is the uniaxial compressive strength of intact rock, *E*_*rm*_ is rock mass deformation modulus, *E*_*i*_ is Young’s modulus for intact rock, *D* is the disturbance factor, *s* and *a* are constants of the HB Criteria. Since no pre-splitting technique was used while heading and benching excavation, there has been some damage to the surrounding rock mass in the roof and walls of the powerhouse cavern. Considering this fact, the value of the disturbance factor (*D*) of 0.6 is used to obtain compressive strength and deformation modulus of the rock mass for the disturbed zone. The ranges of compressive strength and deformation modulus of the rock mass calculated using Eqs. 9 and 10 for both the undisturbed zone (*D = 0*) and disturbed zone (*D = 0.6*) are presented in Fig. 5. As Fig. 5  indicates, the compressive strength and deformation modulus of the rock mass vary greatly upon changes in the GSI value.

### In-situ stress

**Fig. 6:**
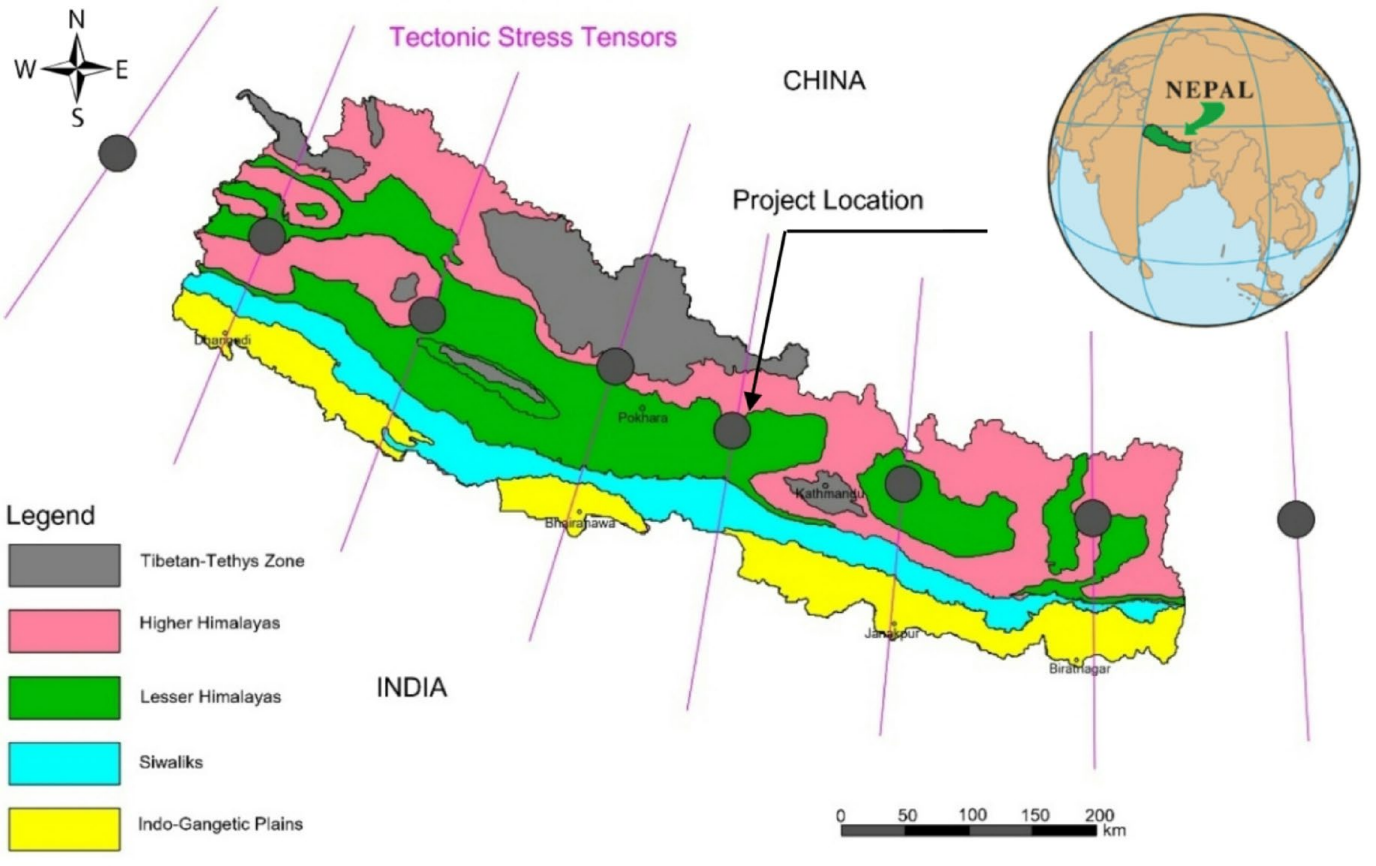
Approximate tectonic stress orientation in the Himalayas and project location.

The in-situ stress in the rock mass can be determined by using hydraulic fracturing, hydraulic jacking, flat jack test, etc. However, due to various difficulties like financial constraints, non-availability of test apparatus, lack of skilled manpower to perform tests, etc., it will not always be possible to perform such tests. The use of numerical modeling to determine in-situ stress in rock mass is indeed a practical alternative when on-site testing data are not available. In the current case, the valley model is simulated using 2D finite element software RS^2^ to compute the magnitude and direction of principal stresses. The valley model considers vertical stress with varying topography and also uses horizontal stress, including the tectonic effect. From the world stress map, the tectonic stress orientation is in the NE-SW in the western Himalayas to almost N-S in the eastern Himalayas. Since the site is located almost in the central part of the Nepal Himalayan range, the tectonic stress orientation is in the direction of NE-SW (Fig. 6).

As seen in Fig. 6, the approximate tectonic stress orientation at the cavern location is N10^0^E. The longitudinal axis of the powerhouse cavern is oriented at N45^0^E and has an angle of 35^0^ with tectonic stress orientation. Since no measured in-situ stress data of the cavern area are available, the horizontal tectonic stress magnitude of 4.5 MPa is adopted following the findings of tectonic stress magnitude for headrace tunnels of the Upper Tamakoshi Hydropower Project (UTHP)^27^ and Parbati-II Hydropower Project^28^respectively, which lie in similar geo-tectonic locations. The laboratory tests provided an average specific weight of the saturated rock samples of 27.8 kN/m^3^ and the Poisson’s ratio of 0.33. Based on these data, the vertical and horizontal stresses are calculated from Eqs. 11 and 12^28^ respectively.

11 $$\:{\sigma\:}_{zz}=\gamma\:H$$

12 $$\:{\sigma\:}_{H}=\frac{\nu\:}{1-\nu\:}\times\:{\sigma\:}_{v}+{\sigma\:}_{tec}.$$

**Fig. 7:**
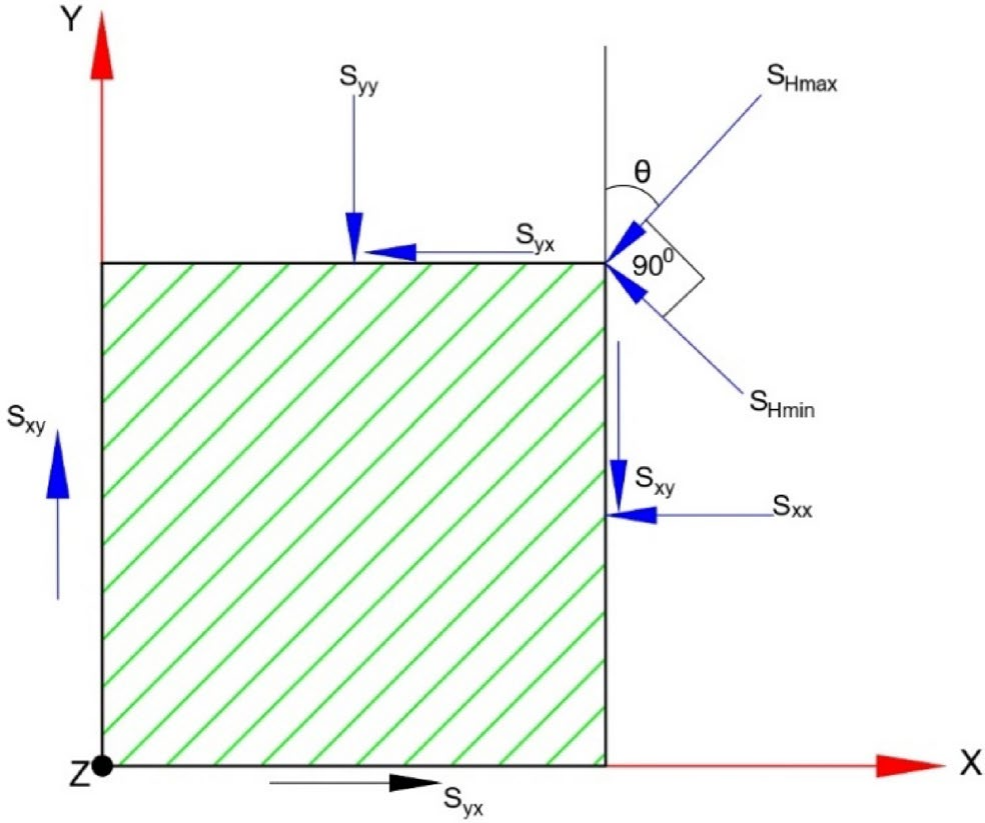
Resolving horizontal stresses in X and Y directions.

In the above equations, γ is the specific weight of the rock, *H* is the overburden height, *ν* is the Poisson’s ratio, and *σ*_*tec*_ is the horizontal tectonic stress in the rock mass. The in-plane horizontal stress, out-of-plane horizontal stress, and horizontal shear stress are given by Eqs. 13–15, respectively. The horizontal stresses in the x and y planes can be resolved as shown in Fig. 7. Also, the maximum total horizontal stress (*S*_*Hmax*_) and the minimum total horizontal stress (*S*_*Hmin*_) are given by Eqs. 16 and 17.

13 $${{\text{S}}_{{\text{yy}}}}={\text{ }}{{\text{S}}_{{\text{Hmax}}}}{\text{co}}{{\text{s}}^{\text{2}}}\uptheta \,+\,{{\text{S}}_{{\text{Hmin}}}}{\text{si}}{{\text{n}}^{\text{2}}}\uptheta$$

14 $${{\text{S}}_{{\text{xx}}}}={\text{ }}{{\text{S}}_{{\text{Hmax}}}}{\text{si}}{{\text{n}}^{\text{2}}}\uptheta \,+\,{{\text{S}}_{{\text{Hmin}}}}{\text{co}}{{\text{s}}^{\text{2}}}\uptheta$$

15 $${{\text{S}}_{{\text{xy}}}}={\text{ }}{{\text{S}}_{{\text{yx}}}}={\text{ sin2}}\uptheta \times \left( {{{\text{S}}_{{\text{Hmax}}}} - {\text{ }}{{\text{S}}_{{\text{Hmin}}}}} \right)/{\text{2}}$$

16 $$\:S_{{Hmax}} = \sigma _{{tec,max}} \: + \:\frac{{\nu \:}}{{1 - \nu \:}} \times \:\sigma \:_{{zz}}$$

17 $$\:S_{{Hmin}} = \sigma _{{tec,min}} \: + \:\frac{{\nu \:}}{{1 - \nu \:}} \times \:\sigma \:_{{zz}}$$

Where, *S*_*yy*_ and *S*_*xx*_ are in-plane and out-of-plane horizontal stresses. *S*_*xy*_ and *S*_*yx*_ are the horizontal shear stresses, *σ*_*tec, max*_ and *σ*_*tec*,min_ are the maximum and minimum tectonic stresses, and *θ* is the angle between the cavern axis and tectonic stress orientation. Table 2 presents the calculated vertical and horizontal stresses in consideration with tectonic stress orientation.

**Table 2:** Input parameters for Valley model.

Input parameters	Symbol	Values	Unit
Overburden	*h*	335	Meters
Poissions ratio	*ν*	0.33	–
Density of rock	*γ*	27.8	kN/m^3^
Tectonic Stress	*σ*_*tec*_	4.5	MPa
Trend of tectonic stress	*θ*_*tec*_	N10^0^E	–
Cavern trend	*θ*_*c*_	N45^0^E	–
Angle between tectonic stress trend and cavern (in-plane) axes	*θ*_*t*_	55	Degrees
Vertical stress due to gravity only	*σ*_*zz*_	9.31	MPa
Maximum total horizontal stress	*S*_*H, max*_	9.09	MPa
Minimum total horizontal stress	*S*_*H, min*_	4.59	MPa
In-plane horizontal stress	*S*_*yy*_	6.07	MPa
Out-of-plane horizontal stress	*S*_*xx*_	7.61	MPa
Horizontal shear stress	*S*_*xy*_ *= S*_*yx*_	2.11	MPa

**Fig. 8:**
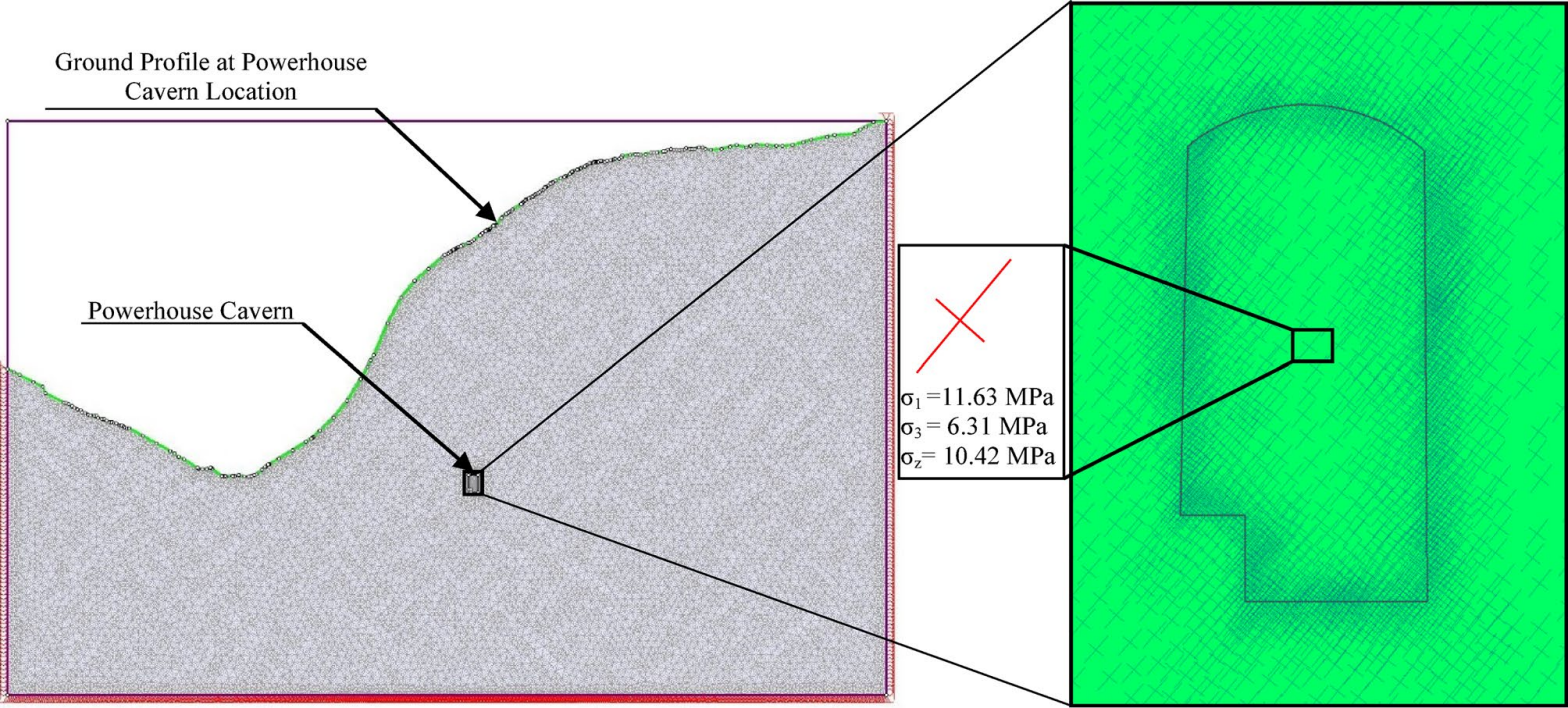
Valley model of the cavern with stress trajectories determined from RS^2^.

The calculated vertical and horizontal stresses are used in the valley model of the cavern to compute the magnitude and direction of major principal stresses (Fig. 8). The magnitude of major principal stress (*σ*_*1*_) and minor principal stress(*σ*_*3*_) are obtained as 11.63 MPa and 6.31 MPa, respectively. Intermediate stress (*σ*_*2*_) is computed as 10.42 MPa. The direction of the major principal stress is approximately 51^0^ counterclockwise with the horizontal axis.

## Stability assessment of cavern with variation in GSI

### Deformation in unsupported cavern for varying GSI

**Fig. 9:**
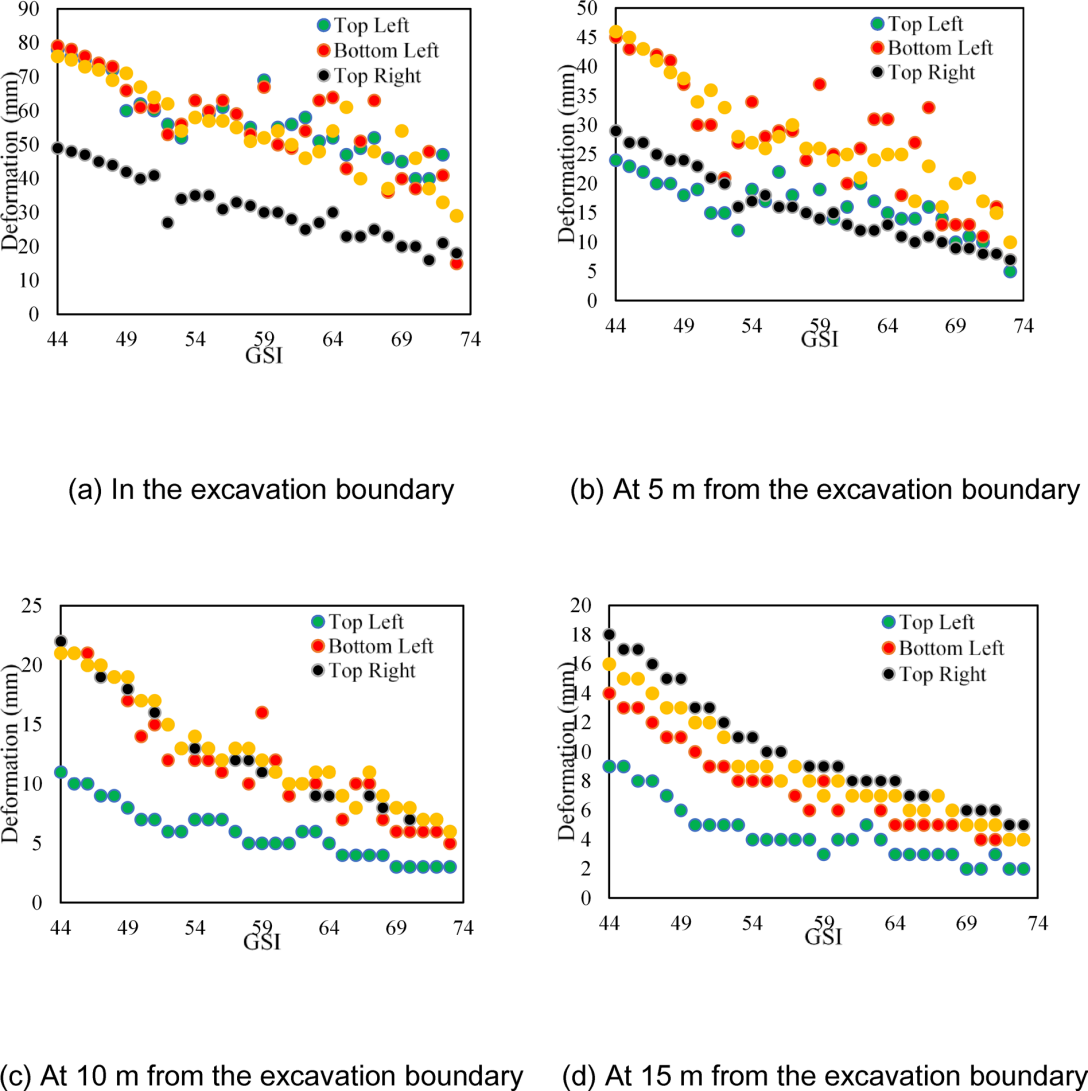
Obtained deformation by 2D numerical modelling at an elevation of 1267.60 m (labelled as top) and at elevation 1259.25 m (labelled as bottom) of unsupported cavern for varying GSI.

**Fig. 10:**
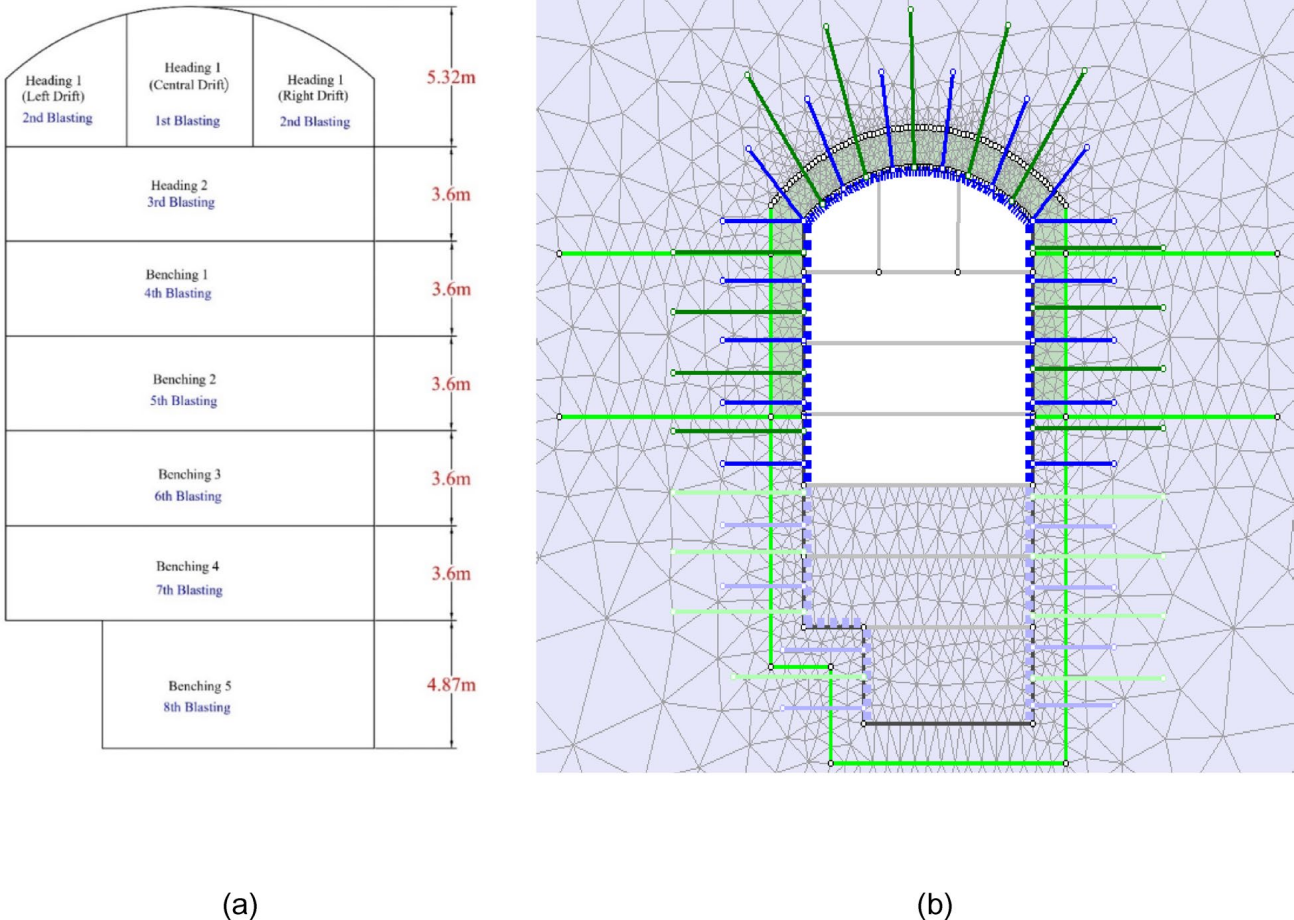
(**a**) Excavation stages of the cavern (**b**) RS^2^ model of excavation before benching 3.

Numerical models of the cavern with varying GSI values are created to determine the development of deformation in the powerhouse cavern. The analysis is performed in the box model of the cavern on RS^2^ with an external boundary of 5 times the cavern size in all directions. Blast-induced excavation damage is considered as a 2 m offset distance, and a disturbance factor (*D*) of 0.6 is applied to the damaged zone. GSI value is varied from 44 to 73, where a residual GSI value of 20 is considered. The deformation obtained for the unsupported cavern at the excavation boundary, at distances of 5 m, 10 m, and 15 m from the excavation surface for various GSI values, is shown in Fig. 9. The deformation values achieved by 2D numerical modelling, shown in Fig. 10, correspond to an elevation of 1267.60 m and an elevation of 1259.25 m, as shown in Fig. 3.

As can be seen in Fig. 9, the deformation decreases when the GSI value increases for the unsupported cavern, which is very logical. However, one should note that the cavern of this size is seldom left unsupported all the way to the bottom. Therefore, the simulated results shown in Fig. 9 are only to demonstrate the sensitivity of GSI values on the overall deformation pattern in the cavern.

### Deformation in supported cavern for varying GSI

As shown in Fig. 3, the actually applied rock support in the cavern consists of 15 to 20 cm thick steel fiber shotcrete in a combination of 25 mm diameter bolts having lengths of 5 and 8 m in an alternative grid with a spacing of 3 m in both directions. The 2D numerical modelling is carried out for the cavern with the actually applied rock support. The cavern was excavated sequentially in 8 stages (Fig. 10). In numerical models, a stress relation of 45% was allowed after each round of blasting. The support was applied in layers to resemble the actual excavation and support conditions. The deformation results achieved through simulation at elevations 1267.60 m and 1259.25 m, respectively, are summarized in Table 3.

**Table 3:** Simulated deformation and tunnel strain at the excavation boundary of cavern for three rock mass quality class.

Q value	Rock type	GSI		Cavern deformation (mm)		Cavern strain (%)	
		Limit	Value	El. 1267.6 m	El. 1259.25 m	El. 1267.6 m	El. 1259.25 m
3–4	Poor	Minimum	44	110.3	157.5	0.79	1.13
		Mean	48	100.8	152.9	0.72	1.09
		Maximum	51	85.3	136.5	0.61	0.98
4–10	Fair	Minimum	52	83.2	137.6	0.59	0.98
		Mean	55	79.9	135.1	0.57	0.97
		Maximum	59	77.8	127.2	0.56	0.91
10–40	Good	Minimum	60	70.1	111.9	0.50	0.80
		Mean	66	71.4	109.6	0.51	0.78
		Maximum	73	51.5	75.0	0.37	0.54

The deformation in the supported cavern is influenced by varying GSI values. However, the deformation rates at elevations 1267.6 m and 1259.25 m differ despite having the same GSI values. This suggests that GSI does not have a consistent relationship with deformation in supported underground structures.

### Cavern stability with the variations on GSI

The moment of resistance (*M*_*RC*_) and the shear resistance (*V*_*RC*_) of fiber-reinforced shotcrete (FRS) can be obtained analytically by using Eqs. 18^29^ and 19^30^.

18 $$\:{M}_{RC}=\frac{{\sigma\:}_{f}\:{t}^{2}\:l}{12}$$

19 $$\:{V}_{RC}=\:{\sigma\:}_{s}\:t\:l.$$

Where *σ*_*f*_ and *σ*_*s*_ are the flexural capacity and shear strength of fiber-reinforced shotcrete (FRS), *t* is the thickness of the shotcrete, and *l* is the critical perimeter. For the supported cavern, the moment and shear forces acting on the shotcrete liner are determined numerically from RS^2^. Considering these moments and shear forces are critical, the internal support pressure generated in the liners can be obtained from Eqs. 18 and 19 by replacing the *M*_*RC*_ and *V*_*RC*_ obtained from RS^2^. Bolts are installed at 3-meter intervals along the plane, resulting in a critical perimeter length of 3 m, with a shotcrete thickness of 20 cm. Considering the two extremes, for a GSI value of 44 and 73, the minimum required support pressure is calculated to be 0.446 MPa and 0.39 MPa, respectively.

The maximum support pressure provided by a ring of shotcrete can also be computed by Eq. 20^31^, and the maximum support pressure provided by the ungrouted bolts can be computed by using Eq. 21^31^.

20 $$\:{P}_{s}^{max}=\frac{{\sigma\:}_{cc}}{2}\left[1-\frac{{\left(R-{t}_{c}\right)}^{2}}{{R}^{2}}\right]$$

21 $$\:{P}_{s}^{max}=\frac{{T}_{bf}}{{s}_{c}{s}_{l}}.$$

Where, *σ*_*cc*_ is the unconfined compressive strength of the shotcrete in MPa, *R* is the external radius of the support and will be the same as the radius of the tunnel, *t*_*c*_ is the thickness of the ring of shotcrete, *T*_*bf*_ is the ultimate load obtained from pull-out test in MN, *s*_*c*_ is the circumferential spacing and *s*_*l*_ is the longitudinal spacing of the bolts.

For the cavern width of 14 m and a 20 cm thick M30 grade of shotcrete, the maximum support pressure that a shotcrete ring may resist is computed as 0.845 MPa. Similarly, for the 25 mm diameter bolts at the longitudinal and circumferential spacing of 3 m, the calculated support pressure is 0.028 MPa. Thus, the maximum possible support pressure that the installed support may carry without failure will be 0.873 MPa. In reality, the support pressure that applied support may resist should be lower than the calculated values since Eqs. 20 and 21 are developed for the circular shape underground excavations, which is not the case for the powerhouse cavern. Therefore, for further calculations, the numerically obtained internal support pressure of 0.446 MPa is considered.

The overall stability of the underground caverns is dependent on the support pressure required to limit the extent of deformation. However, according to Panthi and Shrestha (2018)^32^a significant part of the deformation occurs immediately after the excavation and before the support is applied, which the authors defined as instantaneous closure (*ε*_*IC*_). Over a long period, an underground cavern will experience both instantaneous and time-dependent deformation, which is defined as final closure (*ε*_*FC*_). In this regard, Panthi and Shrestha (2018)^32^ proposed the empirical relationship between the internal support pressure and the tunnel closure expressed by Eqs. 22 and 23.

22 $$\:{\epsilon\:}_{IC}=3065{\left(\frac{{\sigma\:}_{v}\left(1+k\right)/2}{2G\left(1+{P}_{i}\right)}\right)}^{2.13}$$

23 $$\:{\epsilon\:}_{FC}=4509{\left(\frac{{\sigma\:}_{v}\left(1+k\right)/2}{2G\left(1+{P}_{i}\right)}\right)}^{2.09}.$$

Where, *σ*_*v*_ is the vertical stress, *k* is the stress anisotropy factor, *G* is the shear modulus of rock mass, and *P*_*i*_ is the internal support pressure. Equation 23 can be re-formulated to Eq. 24 to find out the shear modulus of rock mass (G) as:

24 $$\:G=\:\frac{{\sigma\:}_{v}(1+k)}{4(1+{P}_{i)})}{\left(\frac{{\epsilon\:}_{FC}}{4509}\right)}^{2.09}.$$

The required shear modulus for rock mass is computed by considering the maximum support pressure of 0.446 MPa and closure listed in Table 4. The available shear modulus for various GSI values is calculated by using the deformation modulus obtained from the generalized Hoek and Diederichs (2006)^26^ relationship and Poisson’s ratio. It is found that the required shear modulus of the rock mass is lower than the available shear modulus for all the rock classes (Table 4), indicating that the cavern is safe and will not face significant instability problems.

**Table 4:** Available and required shear modulus for rock mass.

Q value	Rock type	GSI		Cavern closure (%)		Required shear modulus (GPa)		Available shear modulus (GPa)
		Limit	Value	El. 1267.6 m	El. 1259.25 m	El. 1267.6 m	El. 1259.25 m	
3–4	Poor	Minimum	44	0.79	1.13	1.81	1.52	3.82
		Mean	48	0.72	1.09	1.89	1.55	4.95
		Maximum	51	0.61	0.98	2.05	1.63	5.95
4–10	Fair	Minimum	52	0.59	0.98	2.07	1.63	6.31
		Mean	55	0.57	0.97	2.11	1.64	7.45
		Maximum	59	0.56	0.91	2.14	1.69	9.07
10–40	Good	Minimum	60	0.50	0.80	2.25	1.80	9.49
		Mean	66	0.51	0.78	2.23	1.81	11.92
		Maximum	73	0.37	0.54	2.60	2.18	14.33

## Discussions

For the three different categories of rock mass (i.e., poor, fair, and good), there is a notable variation in the compressive strength of rock mass and deformation modulus (Table 5). The table further shows that the compressive strength of rock mass and deformation modulus for the disturbance factors 0 and 0.6 for the different limits of GSI values for three different categories of rock mass class.

**Table 5:** Rock mass strength and deformation modulus for the different rock quality types.

Q	Rock type	GSI		D = 0		D = 0.6	
		Limit	Value	σ_rm_ (MPa)	E_rm_ (GPa)	σ_rm_ (MPa)	E_rm_ (GPa)
3–4	Poor	Minimum	44	4.73	10.16	2.14	4.14
		Mean	48	6.00	13.17	2.89	5.36
		Maximum	51	7.15	15.83	3.59	6.51
4–10	Fair	Minimum	52	7.58	16.78	3.86	6.94
		Mean	55	9.01	19.81	4.80	8.40
		Maximum	59	11.32	24.13	6.38	10.73
10–40	Good	Minimum	60	11.98	25.24	6.85	11.37
		Mean	66	16.82	31.69	10.47	15.65
		Maximum	73	24.91	38.11	17.11	21.01

As can be seen in Table 5, there is considerable variation in the compressive strength of rock mass and deformation modulus within the same rock mass class category upon variation of GSI values from minimum to maximum.

It should be noted that the GSI is directly linked with parameters *s* and *a*, which are associated to the compressive strength of rock mass calculated using Eq. 9. Hence, a slight variation in GSI value can result in a significant difference in the rock mass strength. Similarly, the deformation modulus of the rock mass calculated using Eq. 10 is also significantly influenced by the changes in GSI within the same rock mass quality class. Therefore, all equations that are linked with GSI and other rock mass classification systems are very sensitive to the same rock mass class category and should be used with care.

Similarly, in rock engineering practices, the MC failure criterion, which is linked with friction angle and cohesion, is widely used. Hoek (2007)^16^ provides the equivalent angle of friction *ϕ*^*’*^ and cohesive strengths *c’* of rock mass by utilizing the parameters associated to HB failure criterion expressed by Eqs. 25 and 26.

25 $$\:{\varphi\:}^{{\prime\:}}={sin}^{-1}\left[\frac{6a{m}_{b}{\left(s+{m}_{b}{\sigma\:}_{3n}^{{\prime\:}}\right)}^{a-1}}{2\left(1+a\right)\left(2+a\right)+6a{m}_{b}{\left(s+{m}_{b}{\sigma\:}_{3n}^{{\prime\:}}\right)}^{a-1}}\right]$$

26 $$\:{c}^{{\prime\:}}=\frac{{\sigma\:}_{ci}\left[\left(1+2a\right)s+\left(1-a\right){m}_{b}{\sigma\:}_{3n}^{{\prime\:}}\right]{\left(s+{m}_{b}{\sigma\:}_{3n}^{{\prime\:}}\right)}^{a-1}}{\left(1+a\right)\left(2+a\right)\sqrt{1+\left(6a{m}_{b}{\left(s+{m}_{b}{\sigma\:}_{3n}^{{\prime\:}}\right)}^{a-1}\right)/\left(\left(1+a\right)\left(2+a\right)\right)}}.$$

**Fig. 11:**
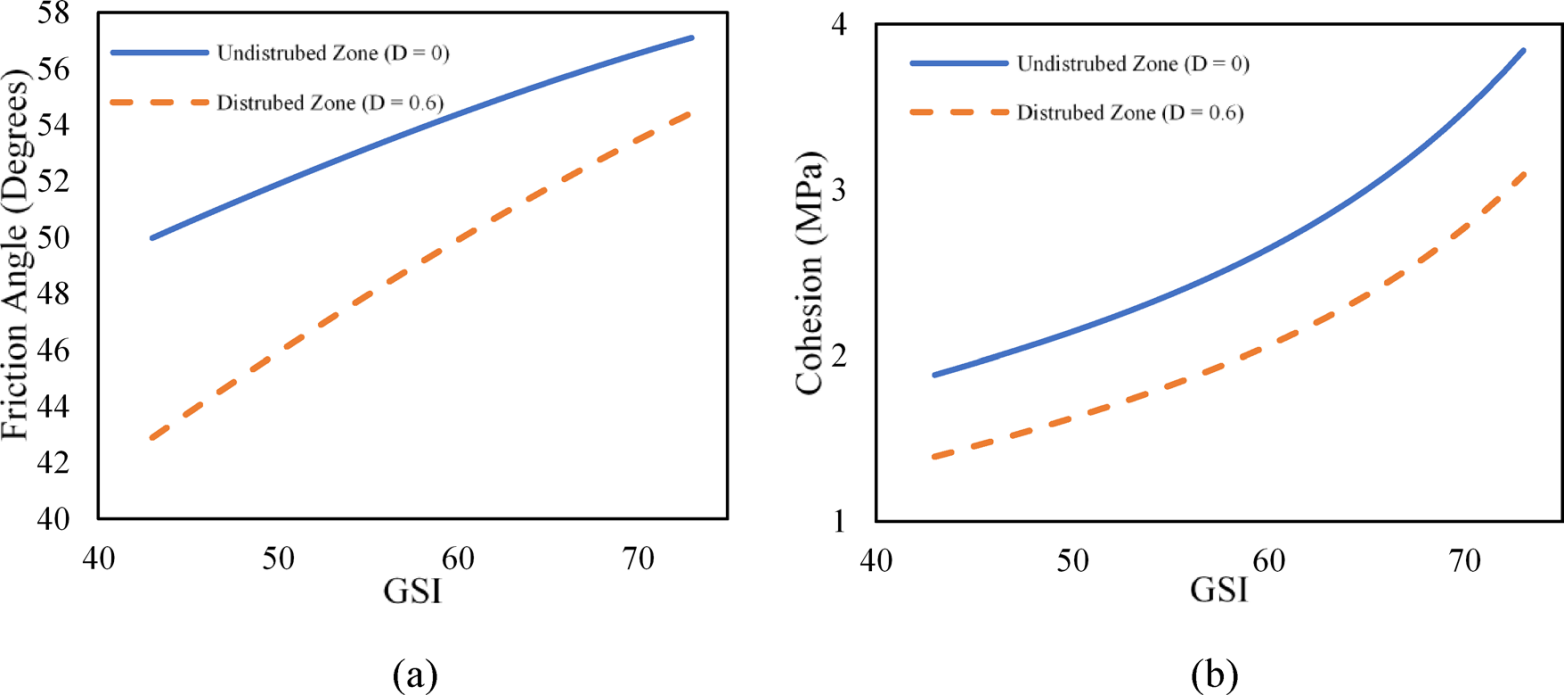
Variation of (**a**) Friction Angle (**b**) Cohesion with respect to GSI.

Following Eqs. 25 and 26, both friction angle and cohesion are linked with constants *m*_*b*_, *s*, and *a*, which are again linked with GSI. This means that, upon variation in GSI values, the angle of friction and cohesion will also vary within the same rock mass class category (Fig. 11).

**Fig. 12:**
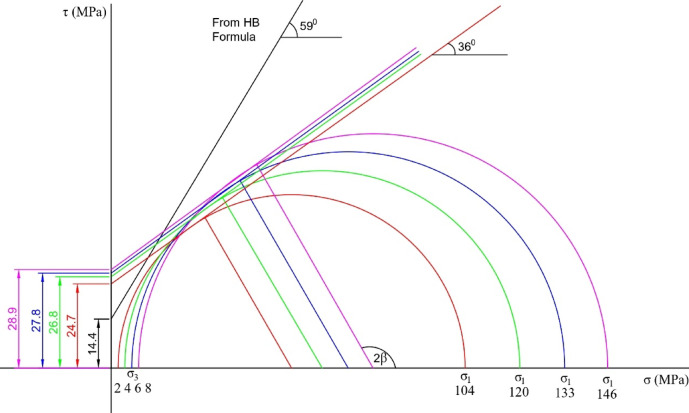
M-C Envelope from multistage triaxial test of intact rock.

The triaxial test was carried out for the intact rock samples of the powerhouse cavern area. Figure 12 shows the MC envelope for different confining pressures. As can be seen in the figure, the average friction angle and cohesion of intact rocks are around 36 degrees and 25 MPa, respectively.

**Fig. 13:**
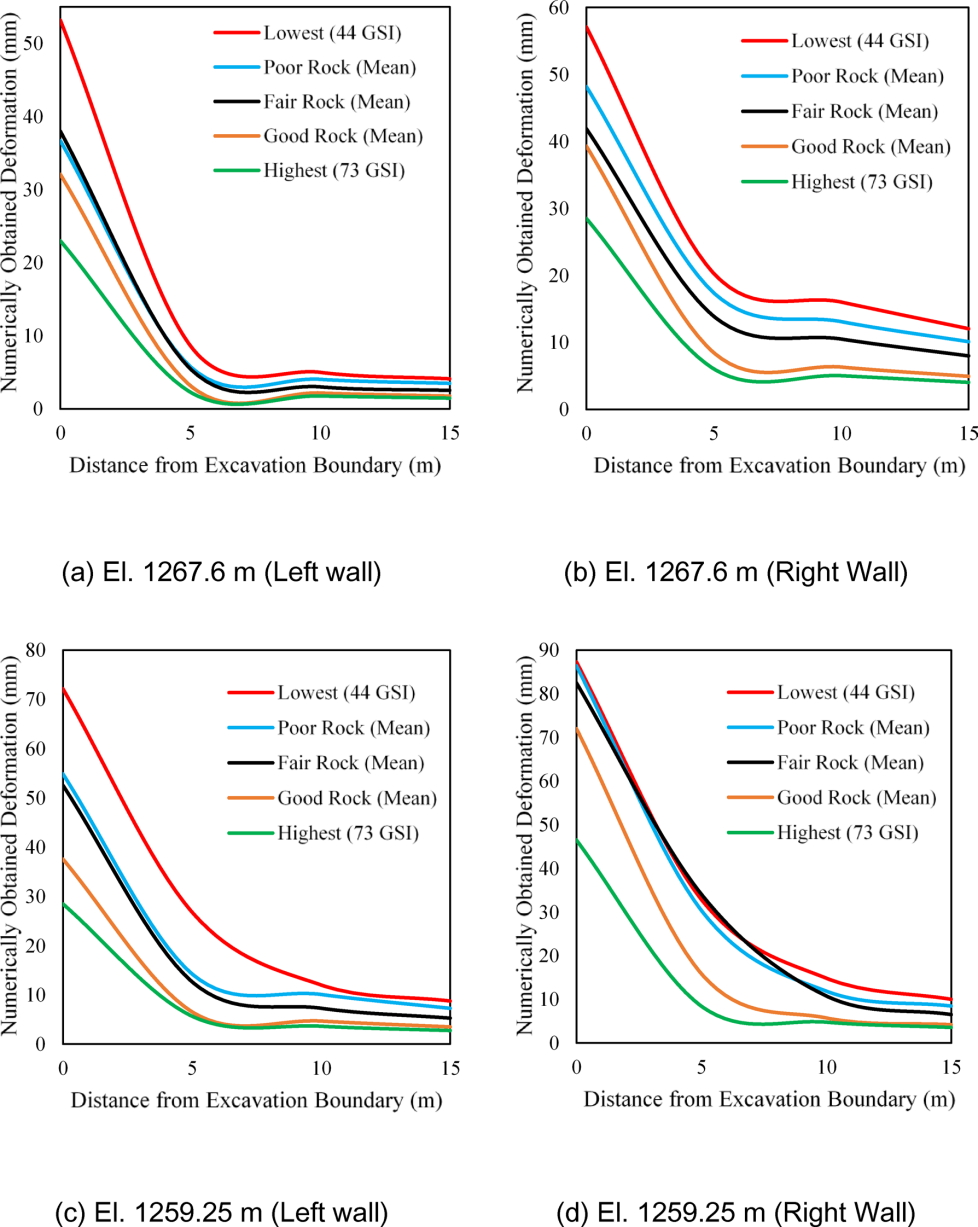
Deformation obtained from the numerical model and extensometer reading.

However, if the same is calculated using Eqs. 25 and 26, the friction angle and cohesion for GSI value 100 (intact rock) will result in 59 degrees and 14 MPa, respectively. This suggests that there is considerable influence of GSI in calculating friction angle and cohesion since HB criteria are dependent on the constraints (*a*,* s*,* m*_*b*_, and *D*) that are calculated using GSI. Thus, if there is a misjudgment in selecting an appropriate GSI, the error will be amplified in the estimates of cohesion and friction angle by the HB relationship^33^. The deformations on the supported cavern with extreme and mean values of GSI for the various rock mass classes are shown in Fig. 13. As seen in Fig. 13, the deformation tends to reduce considerably deep into the rock mass irrespective of the rock mass quality class, which is logical. However, significant deformation variations are still observed along the excavation boundary due to varying GSI, which may impact the final rock support decision.

The application of empirical classification systems such as GSI, RMR, and the Q-system plays an important role in evaluating rock mass quality and guiding the design of underground structures. However, their differing sensitivities to input parameters can lead to contrasting outputs, even when applied to the same rock mass category. The GSI system, which is based on qualitative assessments of rock mass structure and discontinuity of surface conditions, is particularly sensitive due to subjective field evaluations. In addition, the multiple use of GSI values to calculate different parameters of Hoek and Brown failure criteria and frictional properties of the rock mass creates circular loops. This can introduce erroneous estimation of rock mass strength, deformation modulus, and frictional properties of the rock mass. The RMR system offers a more quantitative approach than GSI. However, it may inadequately represent joint interaction and stress conditions, particularly in tectonically disturbed or heterogeneous rock mass. Similarly, Q-system exhibits high sensitivity to parameter changes and may predict a wider range of deformation responses. These variations in sensitivity can substantially influence the assessment of cavern stability and the design of support systems. Hence, a thorough sensitivity analysis is essential for the reliable interpretation of rock mass behavior.

## Conclusions

There is a significant sensitivity in the use of GSI for the same rock mass quality category. This is especially the case when using the HB Failure criterion, where almost all associated parameters are linked to GSI. It is emphasized here that the extent of deformation in the tunnels and caverns is dependent on the overall rock mass quality, rock mass properties, and in-situ stress situation. Specifically, the conclusion of this study can be summarized as:

1. The GSI is linked to the assessment of different rock mass parameters such as compressive strength of rock mass, deformation modulus, friction angle, cohesion etc., creating a loop.
2. If estimated rock mass properties are linked with the numerical values of rock mass classification, such as GSI, it is very likely that the achieved deformation extent may not represent the real ground condition of an underground opening.
3. The estimation of rock mass properties must not only be carried out by using the GSI values, but also by other methods.

Therefore, the authors conclude that it is important to carry out a sensitivity analysis with multiple rock mass classification systems as well as analytical approaches to ascertain the validity of the stability assessment during numerical modelling of underground structures.

## Data Availability

Data used during this study are available from the corresponding author upon reasonable request.
